# An assessment of dental students’ empathy levels in Malaysia

**DOI:** 10.5116/ijme.5259.4513

**Published:** 2013-11-21

**Authors:** Muneer G. Babar, Hanan Omar, Lee P. Lim, Saad A. Khan, Shahid Mitha, Siti F.B. Ahmad, Syed S. Hasan

**Affiliations:** 1 International Medical University, Jalan Jalil Perkasa 19, Bukit Jalil, Kuala Lumpur, 57000, Malaysia; 2 Faculty of Dentistry, University of Malaya, 50603 Kuala Lumpur, Malaysia; 3 The University of Queensland, 20 Cornwell Street, Woolloongabba, 4102, Brisbane, Australia

**Keywords:** Empathy, dental, students, university, Malaysia

## Abstract

**Objectives:**

To examine the validity and reliability of the Jefferson Scale of Empathy-Health Care Provider Student version (JSE-HPS) in a sample of dental students in Malaysia, with the secondary aim of assessing empathy levels in first to final year dental students in public and private universities in Malaysia.

**Methods:**

The JSE-HPS was administered to 582 first to fifth (final) year dental students; 441 were enrolled at two public universities and 141 at a private university in Malaysia. Both descriptive and inferential statistics were performed using SPSS® version 18.

**Results:**

The JSE-HPS demonstrated good internal consistency (Cronbach’s α = 0.70). A three-factor solution emerged and included ‘perspective taking’, ‘compassionate care’ and ‘standing in patient’s shoes’ factors, accounting for 27.7%, 13.9%, and 6.3% of the variance, respectively. The total mean empathy score was 84.11±9.80, where the actual scores ranged from a low of 22.05 to a high of 133.35. Overall, male students (84.97±11.12) were more empathic than female students (83.78±9.24). Fourth-year students were more empathic than students in other undergraduate years, and public university students had significantly higher mean empathy score compared to those enrolled at a private university (84.74 versus 82.13, p=0.001).

**Conclusions:**

This study confirms the construct validity and internal consistency of the JSE-HPS for measuring empathy in dental students. Empathy scores among students vary depending on type of university and year of study. Future studies, preferably longitudinal in design should explore changes in empathy among dental students during progression through undergraduate courses.

## Introduction

Empathy is one of the basic “elements” of good physician-patient relationships,^1,2^ and is often considered an important attribute for professionals in health care.^3^ Empathy was derived from two Greek terms, “em” and “pathos”, meaning ‘feeling into’ and has its origin from the German word “Einfulung”.^4^ Empathy enables health care professionals to identify and understand patient’s experiences, concerns and perspectives.^5^ Empathy is fundamental to the health care provider-patient relationship.^6^ In terms of patient care, empathy is defined as a cognitive attribute that involves an ability to understand the patient’s experiences, pain, suffering, and perspective, combined with a capability to communicate this understanding and an intention to help.^7^ Pedersen (2009) defines empathy succinctly as the “appropriate understanding of the patient”.^8^ Both empathy and sympathy involve sharing,^9^ but the concept of empathy lies in cognitive understanding,^10^ whereas sympathy involves sharing emotions with the patients.^11,12^Previous studies have reported a decline in empathy among undergraduate medical,^13-17^ and dental students,^18,19^ as they progress through their professional education. A longitudinal study by Hojat et al (2009) found no significant change in the first 2 years of medical school but a significant decline in empathy by the third year that continued throughout the students’ medical training.^16^ A longitudinal study by Sherman and Cramer found that empathy levels drop sometime during the second year of dental training and remained low throughout dental school.^19^ While this decline is commonly reproduced in studies, there are still some studies that found senior students as being significantly more empathetic than junior students.^3,19-21^ Some studies linked “erosions” in empathy level with the learning context, the “hidden curriculum”, student difficulties in dealing with stressors in medical education, and poor role modeling in the academic and clinical workplaces.^16,22,23^Due to its vital role in good dentist-patient relationship, the American Dental Education Association (ADEA) listed empathy as the second most important clinical competency for dental training.^24^ Despite the fact that empathy influences adherence to orthodontic treatment,^25^ facilitates patients’ satisfaction with emergency dental care,^26^ and extr- actions, restorations, and endodontic treatments,^27^ decrease dental fears,^28^ and improves treatment outcomes in patients with myofascial pain,^29^ the role of empathy in the dentist-patient relationship has received little attention,^26^ and only a few studies have examined the level of empathy among dental students.^19,30^Several instruments are available to examine empathy level such as Interpersonal Reactivity Index,^31^ The Empathy Scale,^32^ The Emotional Empathy Scale,^33^ and Jefferson Scale of Physician Empathy (JSPE). JSPE is a well-validated, content-specific and context-relevant instrument.^2,11,21^ The Jefferson Scale of Empathy (JSE) exists in two versions, the physician version (HP/Physician version) and the student version.^7^ There are two versions of the JSE student version, one version is for use with medical students (S-version), and other is aimed at health care provider students (HPS version).^7^ In the JSE-HPS version, 13 items from the medical student version (S-version) were modified by replacing “physician” with “health care provider”.^7,34^ For instance, in the medical student version “Physicians should try to think like their patients in order to render better care” was modified to read “Healthcare providers should try to think like their patients in order to render better care.” Other items remained the same, for example “Because people are different, it is difficult to see things from patients’ perspectives.”^34^The generalization of findings to dental schools is uncertain, since the published literature is mainly restricted to medical schools and the physician version (HP version) of JSE.^19,30,35^ The JSE-HPS version has been validated in a sample of health care provider students,^34,36,37^ but its psychometric properties have not yet been established among dental students. The primary aim of this study was to examine the validity and reliability of the student version of JSE-HPS in a sample of dental students in Malaysia, with the secondary aim of assessing empathy levels in first to final year dental students in a public and a private university in Malaysia.

## Methods

### Study design and population

This cross-sectional study was carried out among first to final-year (fifth year) undergraduate dental students using a well-validated, self-administered Jefferson Scale of Empathy-Health Care Provider Student Version (JSE-HPS). In order to gain a general picture of empathy among dental students, public (government-funded) and private university students were included in this study. Data were collected from students enrolled in Bachelor of Dental Surgery (BDS) degree programs at two government-funded universities, University of Malaya (UM) and University Technology Mara (UiTM), and one private university, International Medical University (IMU). One staff member from each university coordinated the distribution and collection of the anonymous questionnaires. The study was approved by the International Medical University Research and Ethics Committee (IMU-REC) and permission to collect data was obtained from the Dean Offices of UM and UiTM.

### The Jefferson Scale of Empathy - (JSPE-HPS)

A Jefferson Scale of Empathy, Health Care Provider Student version (JSE-HPS) was used in this study.^34,36^ The scale was developed by the Jefferson Medical College, and was originally developed for medical students (S-version),^5,7^ and was later modified to be applicable to practicing physicians and other health professionals.^7,11^ The instrument was found to be reliable among medical students and medical residents, respectively.^5^ The psychometric properties of JSE-HPS scale have been reported as satisfactory and the construct validity of the scale has been examined previously.^34,36^ The instrument consists of 20 items answered on 7-point Likert scale which are scored from 1 (strongly disagree) to 7 (strongly agree). Among the 20 questions, 10 negatively worded items in the scale were reverse scored.^3,7,34^ The total score ranges from 20-140; a higher score indicates a behavioral tendency favoring empathic engagement in patient care.^3,7,17,34^

### Sample size and sampling

During the data collection phase, one of the researchers approached each cohort of students to provide information about the study. Questionnaires were posted via courier service to the coordinators at UM and UiTM with a copy of the ethical approval letter, participant information sheets and consent forms. Convenience sampling was used to enroll all the eligible respondents during the study period. Sampling of students from the target population occurred by inviting every second student on the alphabetical class list to complete the questionnaire. Participants were briefed by the researchers before completing the questionnaire. Participation was anonymous and voluntary, with no reward for participation. Researchers were there in person to clarify any doubts from students. The participants were approached after major teaching and learning sessions to obtain higher response rate. Responses from first to final year dental students were collected at the beginning of the semester. The content and the teaching methods remained stable over the period in which the information was collected.

### Statistical analysis

Both descriptive and inferential data analyses were performed using SPSS® version 18 with 0.05 as the level of significance. Descriptive statistics was used to generate summary estimates on the participants by type of university and study year. Frequencies, percentages, mean, and standard deviations were also calculated. Since JSPE-HPS has not been previously used in Malaysia, we conducted a Principal Component Analysis (PCA)^38,39^ to examine the underlying components of JSE-HPS in dental students. In order to achieve a favorable ratio (>10:1) of respondents over instruments items, a minimum of 200 participants were required to conduct factor analysis.^39^ Next we performed Kaiser-Meyer-Olkin test (KMO) to measure sampling adequacy of >0.7.^20^ An Eigenvalue of >1 was used for retaining factors in PCA.^38^ However, potential bias can be introduced by the use of >1 cut-off value,^38^ and therefore we also inspected the Scree plot as a superior factor selection method to determine the appropriate number of factors to retain for rotation.^40^ Bartlett’s test of sphericity was used to measure significant correlations between variables.^20^ The corrected item-total score correlations were also examined. Internal consistency was analysed using Cronbach’s alpha. Independent T-test and one way analyses of variance (ANOVA) including post hoc tests were computed to examine differences in empathy scores related to gender, age and ethnic groups, type of university and year of study. Chi-square test for association and Pearson test for correlation were also applied.

## Results

### Demographic characteristics

A total of 582 students participated in this study. Of the total sample, 441 (75.8%: UM = 246; UiTM = 195) were enrolled at public universities and 141 (24.2%: IMU = 141) at a private university. The majority of the students was female, and out-numbered male students by 2.6:1. Almost 75% of the students enrolled at the public universities were Malay while more than 90% of the students enrolled at the private university were Chinese. Indian respondents accounted for less than 2% of the total. Half the participants were aged between 21-24 years (50%), see Table 1.

**Table 1:** Demographic characteristics of the study participants (n=582)

Variables	Overall n (%)	Public University n (%)	Private University n (%)	Association (p-value)
Gender				
Male	161 (27.70)	100 (17.20)	61 (10.50)	p = 0.001
Female	421 (72.30)	341 (58.60)	80 (13.70)	
Age group				
18-20	275 (47.30)	220 (37.80)	55 (9.50)	p = 0.052
21-24	291 (50.00)	211 (36.30)	80 (13.70)	
25-28	16 (2.70)	10 (1.70)	6 (1.00)	
Ethnic group				
Malay	333 (57.20)	329 (56.50)	4 (0.70)	p = 0.001
Chinese	231 (39.70)	100 (17.20)	131 (22.50)	
Indian	10 (1.70)	5 (0.90)	5 (0.90)	
Others	8 (1.40)	7 (1.20)	1 (0.20)	
Year of study				
Year 1	167 (28.70)	118 (20.30)	49 (8.40)	p = 0.006
Year 2	157 (27.00)	111 (19.10)	46 (7.90)	
Year 3	107 (18.40)	86 (14.80)	21 (3.60)	
Year 4	66 (11.30)	50 (8.60)	16 (2.70)	
Year 5	85 (14.60)	76 (13.10)	9 (1.50)	

### Principal Component Analysis

A 20 items of JSE-HPS were entered into iterated PCA with Kaiser Normalization. The KMO test of sampling adequacy was applied prior to factor extraction, which resulted in overall index of 0.90, suggesting that the sample was adequate for factor analysis. The Bartlett’s test for sphericity showed that the inter-correlation matrix was factorable (Chi-Square (190) = 3511.7, p<0.001). Inspection of the corresponding Scree plot and identification of an ‘elbow’ point after which the inclusion of additional factors does not result in substantial gains in ‘variance explained’ yielded the existence of at least three factors, with eigenvalues more than one. Based on the plot of the eigenvalues that leveled off after the third factor, a 3-factor solution was selected. The loadings of individual items on these three factors are presented in Table 2.

**Table 2:** Summary of Factor Analysis and corrected item-total score correlations of the JSPE-HPS administered to 582 dental students

Items* (sequence in scale)	Rotated factors coefficients		
	Perspective taking	Compassionate care	Standing in patient’s shoes
1. Health care providers' understanding of the emotional status of their patients, as well as that of their families is one important component of the health care provider – patient relationship. (Q16)	0.717	0.014	-0.037
2. Understanding body language is as important as verbal communication in health care provider - patient relationships. (Q4)	0.695	-0.294	-0.047
3. Patients feel better when their health care provider understands their feelings. (Q2)	0.690	-0.187	-0.059
4. Health care providers should try to stand in their patients' shoes when providing care to them. (Q9)	0.687	-0.098	0.095
5. Health care providers should try to think like their patients in order to render better care. (Q17)	0.675	-0.034	-0.172
6. Health care providers should try to understand what is going on in their patients' minds by paying attention to their non-verbal cues and body language. (Q13)	0.674	-0.327	0.130
7. Patients value a health care provider's understanding of their feelings which is therapeutic in its own right. (Q10)	0.670	-0.308	0.057
8. A health care provider's sense of humour contributes to a better clinical outcome. (Q5)	0.631	-0.241	0.072
9. I believe that empathy is an important factor in patients' treatment. (Q20)	0.602	-0.172	0.136
10. Empathy is a therapeutic skill without which a health care provider's success is limited. (Q15)	0.458	0.173	-0.022
11. Health care providers should not allow themselves to be influenced by strong personal bonds between their patients and their family members. (Q18)	0.377	0.090	0.183
12. I believe that emotion has no place in the treatment of medical illness. (Q14)	-0.108	0.752	0.035
13. Patients' illnesses can be cured only by targeted treatment; therefore, health care providers' emotional ties with their patients do not have a significant influence in treatment outcomes. (Q11)	-0.125	0.728	0.052
14. Asking patients about what is happening in their personal lives is not helpful in understanding their physical complaints. (Q12)	-0.157	0.728	0.076
15. Attention to patients' emotions is not important in patient interview. (Q7)	-0.110	0.722	-0.074
16. Attentiveness to patients' personal experiences does not influence treatment outcomes. (Q8)	-0.090	0.721	0.165
17. I do not enjoy reading non-medical literature or the arts. (Q19)	0.007	0.589	0.050
18. Health care providers’ understanding of their patients’ feelings and the feelings of their patients’ families do not influence treatment outcomes. (Q1)	-0.132	0.540	0.226
19. It is difficult for a health care provider to view things from patients' perspectives.(Q3)	0.093	0.074	0.771
20. Because people are different, it is difficult to see things from patients' perspectives. (Q6)	0.044	0.131	0.730
Cronbach’s alpha	0.84	0.82	0.40
Percent of variance (%)	27.7	13.9	6.3

*Items were listed in a descending order of magnitude of factor coefficients by factor and the sequence of the items were put in the form of numbers in parentheses.

The three underlying factors were labeled as “perspective taking”, “compassionate care” and “standing in patient’s shoes”. Eleven items had the highest factor coefficients (≥0.35) on the first extracted factor, which accounted for the largest proportion of the variance before rotation (27.7%). Seven items under “compassionate care” and 2 items under “standing in patient’s shoes” had significant factor loadings (>0.35), accounted for 13.9%, and 6.3% of the variance, respectively. The total variance explained by the three dimensions of empathy was 47.8%. Cronbach’s alpha values were acceptable for all three identified factors, and ranged from 0.40 for factor 3 to 0.84 for factor 1 and 0.82 for 2. The overall Cronbach’s alpha value of the scale was 0.70 which indicates acceptable, satisfactory reliability. An analysis of the individual JSE-HPS items showed that respondents tended to answer all items.

### Comparisons of empathy levels

**Figure 1:**
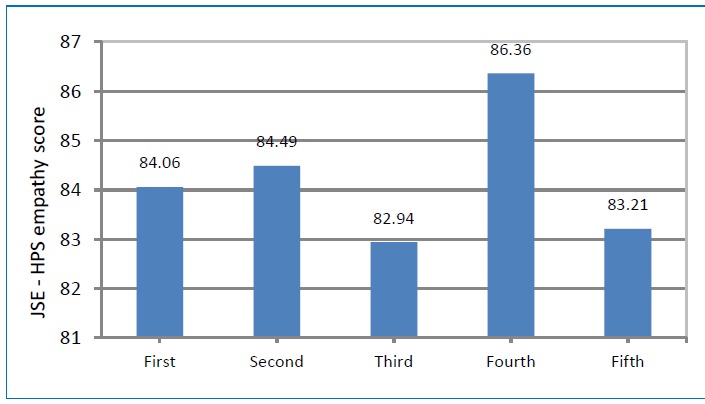
Mean empathy levels by year of study in dental schools in Malaysia

Table 3 summarized the descriptive statistics of the study. The mean empathy score for 582 students was 84.11±9.80. The total actual scores ranged from a low of 22.05 to a high of 133.35 (possible score range: 20 to 140). Male students had slightly higher mean empathy score (mean=84.97, SD= 11.12) compared to female students (mean=83.78, SD= 9.24). Students aged between 25 and 27 years, and students of Malay origin had higher scores compared to students aged between 18 and 24 years and students of other ethnic origins (Table 4), but differences were insignificant. Students enrolled at public university had significantly higher mean empathy score compared to students enrolled at private university (84.74 versus 82.13, p<0.001). Third-year dental students had the lowest mean empathy score (mean= 82.94, SD=9.88) compared to students in other study years. Whereas students in fourth-year had the highest empathy level compared to other study years (mean=86.36, SD= 13.35) (Figure 1). However, there was no significant difference in empathy scores between the year levels of study; similarly, post hoc testing did not demonstrate any statistically significant difference when comparing the difference between each of the study years.

**Table 3:** Descriptive statistics for the JSE-HPS in dental students (n = 582)

Items	Values
Score, Mean (SD)	84.11 (9.80)
Score, Median	83.30
25th Percentile Score	78.25
50th Percentile (Median) Score	83.30
75th Percentile Score	88.35
Possible Score Range	20-140
Actual Score Range	22.05-133.35
Alpha Reliability Coefficient	0.70

**Table 4:** Overall mean scores of JSPE-HPS measures (n = 582)

Variables	N	Mean	SD	Difference p-value
Gender				
Male	161	84.97	11.12	NS
Female	421	83.78	9.24	
Age group				
18-20	275	84.55	9.54	NS
21-24	291	83.54	9.96	
25-28	16	86.81	11.14	
Ethnic group				
Malay	333	85.11	10.65	0.019
Chinese	231	82.66	8.09	
Type of university				
Public	441	84.74	10.48	0.001
Private	141	82.13	6.97	
Year of study				
Year 1	167	84.06	8.93	NS
Year 2	157	84.49	9.67	
Year 3	107	82.94	9.88	
Year 4	66	86.36	13.35	
Year 5	85	83.21	8.08	

After stratification by type of university, we found that students of Indian origin enrolled at public universities (86.19 ± 11.86), and students of Chinese origin enrolled at private university (82.40 ± 6.61) had the highest mean empathy score compared to students of other ethnic origins enrolled at both, public and private universities (Figure 1, Table 5). Students aged between 21 and 24 years in public universities (84.86 ± 10.03), and students aged between 25 and 28 in private university (84.83 ± 6.06) had highest empathy mean scores compared to students in other age groups. Students in the fourth-year in public universities (85.24 ± 9.81), and third-year students in private university (83.76 ± 7.57) had the highest mean empathy score compared to students in other study years.

**Table 5:** Mean scores of JSPE-HPS measures, by public and private universities (n = 582)

Variables	Overall mean score (SD)	Public Uni mean score (SD)	Private Uni mean score (SD)
Gender			
Male	84.97 (11.12)	84.76 (9.93)	82.60 (7.12)
Female	83.78 (9.24)	84.73 (10.69)	81.77 (6.88)
Age group			
18-20	84.55 (9.54)	84.81 (10.89)	81.20 (6.83)
21-24	83.54 (9.96)	84.86 (10.03)	82.57 (7.11)
25-28	86.81 (11.14)	82.12 (9.00)	84.83 (6.06)
Ethnic group			
Malay	85.11 (10.65)	85.07 (11.73)	79.83 (9.68)
Chinese	82.66 (8.09)	84.12 (8.31)	82.40 (6.61)
Indian	81.99 (10.92)	86.19 (11.86)	77.10 (12.97)
Year of study			
Year 1	84.06 (8.93)	85.01 (12.00)	80.97 (6.07)
Year 2	84.49 (9.67)	84.76 (8.76)	82.75 (7.94)
Year 3	82.94 (9.88)	84.53 (10.98)	83.76 (7.57)
Year 4	86.36 (13.35)	85.24 (9.81)	81.61 (7.14)
Year 5	83.21 (8.08)	83.11 (8.18)	82.38 (4.12)

## Discussion

The main objectives of this study were to describe and summarize the psychometric properties of JSE-HPS, including its internal consistency and factor structure and to assess the empathy level among dental students of public and private universities in Malaysia. The mean empathy score of 84 in this study is much lower than the average empathy scores of 103 – 117 reported by previous studies among medical,^17,21,40-42^ and dental students,^19^ using S-version and HP-version of JSE. However, our mean empathy score is comparable to the average empathy scores of 78 – 90 reported in studies among dental students where HP-version of JSE was used.^30,35^ The JSE-HPS has been administered in a cross-sectional manner with pharmacy and nursing students previously,^34,36^ showing a mean empathy score of 111. Again this empathy score is much higher compared to 84 in our study.

The total variance explained by the three dimensions of empathy instrument (47.9%) is higher than findings reported in previous studies among medical students with S-version,^40,43^ and pharmacy students with HPS-version.^34^ In our factor analysis, there were three underlying principal factors identified in the JSE-HPS instrument, namely “perspective taking”, “compassionate care”, and “standing in patient’s shoes”.

Perspective taking describes the understanding of patient’s concerns while compassionate care was labeled to explain the association of feeling and emotion with empathy understanding^34^ and is the core ingredient of empathy, while compassionate care is considered as an important aspect for healthcare provider-patient relationship.^11,19,21,34,44^ “Standing in patient’s shoes” indicates an ability to comprehend and reflect patients’ concerns.^2,45^ These factors are similar to the ones reported in previous studies among nursing students,^36,37^ supporting the construct validity of this instrument for dental students.^11,40,43,44^ However a study conducted in a pharmacy school in the United States reported only two underlying components,^34^ namely perspective taking and compassionate care. The reason being the authors did not follow Kaiser’s suggestion to retain factors with an eigenvalue greater than one,^46^ instead followed Velicer and Fava method, which suggests a minimum of 3 items per factor for a stable structure.^47^Consistent with previous study by Grace et al,^48^ our results showed that male students obtained a higher total mean empathy score than female students. Most studies report that women are more empathic than men.^3,11,19,20,21,34^ and some have argued that empathy is a feminine trait and that females are more receptive emotional signals.^3,11^ Two studies explained their findings in terms of the evolutionary theory of parenting as women tend to display more care-giving attitudes compared to men.^11,21^ No significant difference between students in different age groups was found and, as such, the results overall show the extent of empathy to be more similar than different across the various age groups.

Malaysia is a multi-racial country with three distinct ethnic groups. Malays are the dominant ethnic group, followed by Chinese and Indians in Malaysia. Overall Malay students had higher empathy level compared to Chinese and Indian students in our study. However after stratification by type of university, we found that students of Indian origin in public and students of Chinese origin in the private university had the highest mean empathy score. This difference in empathy level could be a result of their different cultural values, religious beliefs or traditions.^40^ It has been reported earlier that cultural differences, ethnicity, religious beliefs, and sex stereotyping may lead to empathy score disparity,^21,44^ and can also influence empathic engagement during clinical encounters.^40^ Interestingly, public university students were more empathic than private university students in our study. There could be several reasons to explain this finding. For instance, public universities in Malaysia are associated with their own teaching hospitals which allow students to have more frequent visits to hospitals and patients, resulting in increased exposure to patients which may improve the empathy level. Nevertheless, more research should be carried out to identify reasons for this difference, especially in developing countries for which there is a paucity of literature.

Few studies have investigated potential differences in empathy between students from different study year in dental schools, suggesting significant gaps in the literature,^19,30^ Students in the fourth-year had higher mean empathy score compared to students in other study years, whereas students in the final-year had lowest empathy score in our study. The increased levels of empathy in second-year and fourth-year could be attributed to lectures, role-playing or communication skills sessions completed recently. It is argued that even the informal curriculum can also have a significant impact.^49^ Beattie et al found significant increase in empathy level measured before and after an early analytical exposure to behavioural sciences and the clinical encounter.^30^ Empathy levels appear to drop during the third-year of dental training when patient contact increases. This decline in student empathy appears to be a common phenomenon emerging in the literature.^3,15,17,19^ As empathy is a core “ingredient” of good health care professional-patient relationship,^1,2^ improving students’ empathy is one of the important tasks of medical education.^44^ However, empathy is generally only taught in a context where it is not formally evaluated and is rarely integrated into clinical teaching and learning.^30^

### Limitations of the study

This study had several limitations that may affect its generalization. This study was completed early in the academic year, and students’ responses may be based on learning experiences of the previous year. Our assessment of empathy level was based on self-report measures of a validated instrument, and not on the actual behaviours; observational methods such as the History-taking Rating Scale (HRS) could be used with JSE-HPS to measure empathy level in dental students. Lastly, our study was cross-sectional in design which did not allow for a baseline assessment or tracking changes in empathy level across the year levels of the program.

## Conclusion

The scale appears to be reliable based on good internal consistency, supporting the construct validity of this instrument for dental students. The empathy level of students who participated in this study was much lower to the average empathy level reported by previous studies. Overall males and students of Malay origin were more empathic than females and students of other ethnic origins. Fourth-year students were more empathic than dental students in other undergraduate years with the lowest levels measured among students in their final (fifth) year. Dental students enrolled at the public universities were significantly more empathic than dental students enrolled at a private university. Given the importance of empathy in maintaining and improving the dentist-patient relationship, continued research in more diverse dental student populations could have important implications in the education and training of dental students. Future studies, preferably longitudinal in design should explore changes in empathy level in dental students, particularly in developing countries.

## 

### Conflict of Interest

The authors declare that they have no conflict of interest.
